# Tunable Plasmon‐Enhanced Wide‐Angle Terahertz Perfect Absorption via Lorentz Media

**DOI:** 10.1002/nap2.70150

**Published:** 2026-05-27

**Authors:** Yuxuan Zhou

**Affiliations:** ^1^ College of Science & Engineering University of Glasgow Glasgow UK

## Abstract

Perfect absorption (PA) is central to spectroscopic sensing and integrated photonics. Existing mechanisms such as resonant metamaterials, multilayer interference cavities, and coherent or critical coupling schemes have achieved PA. Despite progress, realizing PA with simple structure and high integration at wide angles remains challenging. In this work, the dispersion is innovatively regulated by a composite matching mechanism of phase and amplitude, with a Lorentz‐type dispersive dielectric providing the frequency‐dependent phase response and controlled loss and a distributed Bragg reflector (DBR) supplying cavity feedback, together they realize wide‐angle terahertz (THz) PA in a Tamm‐type structure. Unlike traditional PA approaches that require thick dielectric layers and exhibit angular drift, this approach yields absorption above 92% for incidence angles up to 80° with a subwavelength top layer (∼λ/22). By tuning the Lorentz parameters and the dielectric‐layer thickness, we reconfigure the absorption spectrum without altering the remaining stack, enabling multichannel operation with less than 5.7% frequency shift under oblique incidence. The mechanism combines Lorentz‐driven phase control with cavity feedback and provides a route to minimal patterning and tunable integration for on‐chip photonics.

## Graphical abstract

A planar graphene‐Lorentz‐DBR structure realizes wide‐angle terahertz perfect absorption through a composite phase‐amplitude matching mechanism. The Lorentz dielectric provides dispersive phase compensation and controlled loss, whereas graphene supports dissipative coupling, enabling high absorption up to 80° incidence and multichannel spectral tunability without complex patterning.

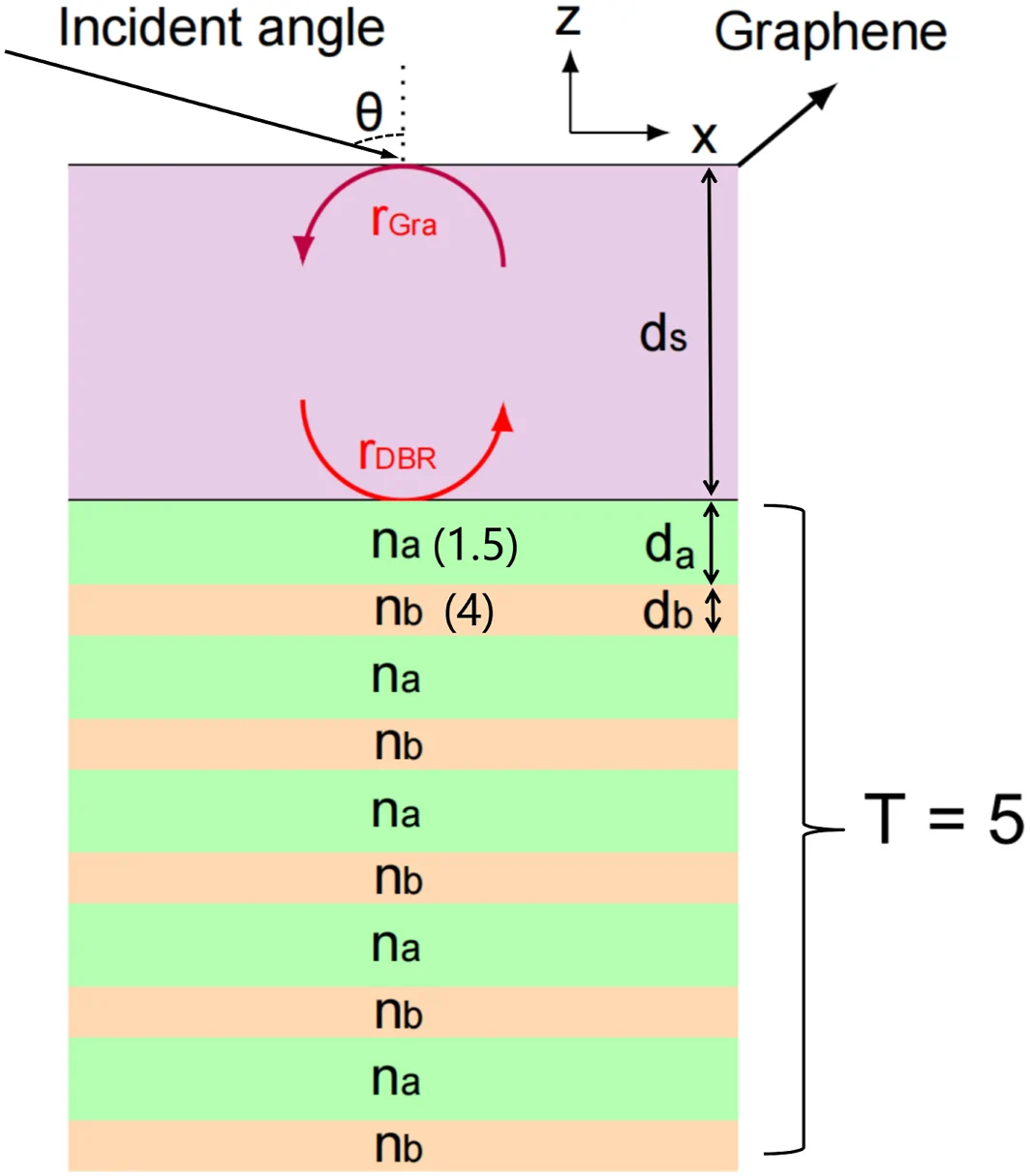

## 1 Introduction

Absorption controls energy transfer between electromagnetic fields and matter, so it sets detection limits in spectroscopy, governs thermal emission, and informs the design of integrated photonics [1, 2]. Perfect absorption functions as a benchmark for coupling efficiency because it aligns impedance, material loss, and modal confinement to drive energy into the intended channel [3, 4, 5]. Existing approaches follow distinct physical principles. Metamaterial‐based absorption employs patterned metallic or dielectric resonators on subwavelength scales to induce electric and magnetic dipole resonances [6]. These resonances tune the effective impedance to match free space, which minimizes reflection and produces strong narrowband absorption [7]. Interference‐based absorption, including Salisbury screens and Fabry–Perot cavities, uses stacked dielectric or lossy layers to control constructive and destructive interference [8]. By adjusting layer thickness, phase delay, and interface reflection coefficients, these systems reach unity absorption at designed frequencies [9]. A third route realizes coherent perfect absorption through critical coupling, where incident beams arrive with specified amplitudes and phases so that coherent superposition directs power into loss channels [10].

These categories are not exclusive; metamaterial layouts can incorporate interference effects and, under counter‐propagating illumination, implement coherent perfect absorption [11, 12]. These approaches provide complementary design variables, including geometric patterning in metamaterials, thickness and refractive‐index contrast in stratified media, and phase control in interferometric layouts, which tune spectral selectivity, angular response, and structural complexity in practice [13, 14]. Meanwhile, structures that incorporate two‐dimensional materials add material‐level tunability of absorption while retaining planar processing and minimal patterning [15]. In a Tamm cavity, the tunable surface conductivity of the two‐dimensional layer cooperates with interference in the multilayer cavity to deliver multichannel response and no‐angle dispersion within a defined design range in subwavelength planar structures while avoiding extensive lithographic patterning [16, 17].

Application demands have shifted perfect absorption toward multichannel spectra, no‐angle dispersion within a defined design range, subwavelength optical thickness, electrical tunability, and fabrication sequences compatible with standard process flows [18, 19]. In this setting, perfect absorption suppresses reflection and transmission and concentrates electromagnetic energy in the active region, which strengthens light–matter interaction and reduces electromagnetic noise. Current studies therefore seek mechanisms that combine spectral selectivity with angular tolerance while keeping the stack thin and avoiding complex patterning [20]. Recent plasmon‐enhanced researches have also shown that hybrid resonant cavities and metal coupled nanostructures can improve field localization and optical response in sensing applications. For example, Li et al. designed a 3D hybrid Ag/MOF multi‐plasmon resonant cavity system for SPR sensing, in which cavity plasmon coupling enhanced hotspot formation and electric field intensity, whereas Liu et al. reported Au‐conjugated lanthanide nanoprobes that combined optical temperature sensing with photothermal control, showing that plasmon coupled nanostructures can also support integrated optical monitoring functions [21, 22]. Although these studies address different structures and applications, they further illustrate the significance of plasma coupling field enhancement in optical devices.

Two‐dimensional materials also support this direction because tunable surface conductivity and carrier density enable strong field confinement, dynamic modulation, and straightforward integration on dielectric mirrors or waveguide platforms [23, 24]. Within these developments, Tamm‐type perfect absorption has received broad attention because a planar conductive layer coupled to a distributed Bragg reflector forms a cavity that matches impedance with films, yields narrowband selectivity, and maintains compact thickness [25, 26]. For example, Orojloo et al. showed that by adjusting the thickness and material composition of each layer as well as the graphene chemical potential, one can control the resonance frequencies and the number of absorption channels, achieving near‐unity absorption at both normal and oblique incidences [27]. Studies on graphene absorbers have also demonstrated that surface plasmon resonance designs can support both multipeak absorption and high angular tolerance. For example, Ai et al. reported a four peak graphene absorber with weak sensitivity to incident angle, indicating that graphene resonant structures can maintain stable multi‐channel absorption [28]. Similarly, Tavana et al. proposed a graphene‐based hyperbolic metamaterial sandwiched between photonic crystals to realize tunable ultra‐narrowband THz absorption with high quality factors and wide‐angle and polarization‐insensitive performance through hybridization of Tamm modes and graphene anisotropy [16]. These studies illustrate the versatility of Tamm‐type configurations and their compatibility with planar fabrication processes. Despite these advantages, practical designs still face trade‐offs, including residual angular dispersion, sensitivity to thickness tolerances, the need for multiple DBR periods to preserve a wide stop band, and limited flexibility for true multichannel operation without additional structural burden.

Recent work in the infrared and visible regimes shows that Lorentz‐type dispersive dielectrics can enhance absorption by leveraging oscillator resonances [29, 30]. The Lorentz oscillator response links a resonant increase in the imaginary permittivity to a steep frequency‐dependent phase response, which provides two coupled controls: loss for amplitude matching and dispersive phase for cavity compensation [31]. Near resonance, the rapid variation of the reflection phase supplies optical phase without added physical thickness; this helps satisfy the round‐trip condition at oblique incidence and reduces angular drift of the absorption peak within the designed range [32, 33]. Strong light–matter coupling at the material resonance enables bandwidth control [34]. By calibrating oscillator strength, damping, and background permittivity, one can set linewidth and quality factor; with multiple oscillators, several narrow bands can coexist in a single planar stack [35, 36]. Because the control resides in dispersion rather than geometric patterning, the stack remains planar and subwavelength and integrates naturally with cavity feedback. Published studies on Tamm‐cavity perfect absorption mainly employ metallic or graphene interfaces, and wide‐angle behavior is typically pursued through alternative dispersion‐engineering routes such as hyperbolic media [25, 37]. We do not find reports that use a Lorentz‐type dispersive dielectric as an explicit phase‐compensation element in a planar Tamm cavity while targeting no‐angle dispersion and multichannel response without complex manufacturing steps. These requirements are particularly critical for practical applications in sensing, imaging, and tunable detection, where performance must remain consistent under varying incident angles and spectral demands [38, 39].

In this context, we insert a Lorentz medium into the Tamm cavity so that its dispersive phase response and controlled loss provide phase‐amplitude matching, which supports no‐angle dispersion within a defined range while preserving spectral selectivity. Building on this mechanism, we develop a phase‐amplitude matching framework in which the Lorentz phase response and material loss jointly set the cavity round‐trip condition and the external impedance. We implement a planar stack comprising graphene, a Lorentz dielectric, and a distributed Bragg reflector that achieves perfect absorption with no‐angle dispersion within a defined design range, multiple spectral channels, and subwavelength optical thickness while keeping the process flow simple. Building on this, we explore how the absorption spectral response can be tuned by varying the dielectric thickness and photonic‐crystal parameters. Together, these results show that the Lorentz‐type dielectric that have frequency dispersion provide the phase and loss needed by the matching condition and underscore the potential for enabling compact, tunable, and wide‐angle absorption.

## 2 Results and Discussion

### 2.1 General Concept of Lorentz THz Perfect Absorption

Prior implementations of PA relied on geometric patterning, fixed‐thickness interferometric stacks, or externally set illumination phases. We pursue a material‐based route in which a Lorentz dielectric provides both the loss and the frequency‐dependent phase required by the matching conditions. Tying amplitude balance and phase closure to the dispersion of the medium, rather than added structure, supports fewer DBR periods, subwavelength thickness, reduced angular drift within a defined range, and multichannel control through tuning of the Lorentz parameters and the top‐layer thickness.

To verify wide‐angle THz PA predicted by the phase‐amplitude matching framework, we designed a Lorentz perfect absorber (LPA) consisting of a graphene interface, a dispersive dielectric layer (the top layer) characterized by Lorentz‐type permittivity, and a one‐dimensional photonic crystal (1DPC), as shown in Figure 1a. In contrast, the top layer of a traditional perfect absorber (TPA) is made of a non‐dispersive and lossless dielectric material, as shown in Figure 1d. We choose the Thz range because polar dielectrics exhibit pronounced Lorentz dispersion and graphene offers tunable sheet conductivity; together supply the phase and loss required to the phase‐amplitude matching conditions in LPA. These conditions depend on impedance balance and round‐trip phase rather than absolute wavelength, so the same design logic extends to other bands when materials provide analogous dispersive phase and dissipation.

**FIGURE 1:**
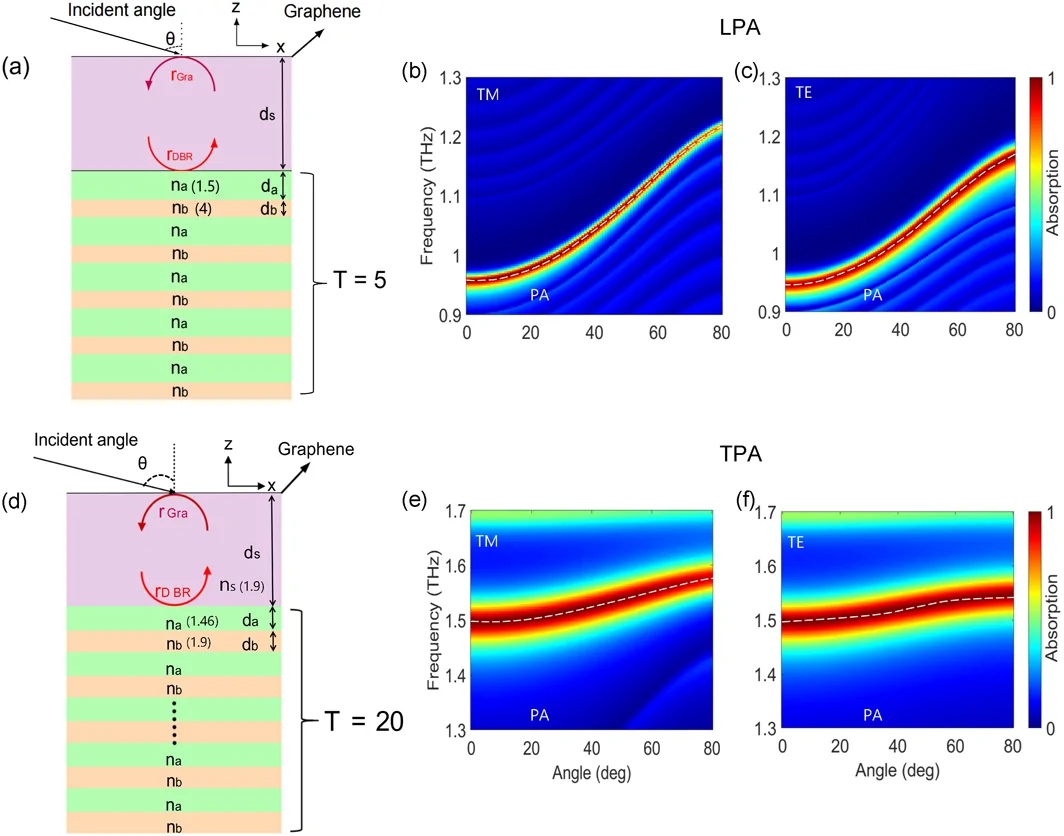
(a) Schematic of LPA, composed of graphene‐dielectric‐1DPC hybrid structure. A Lorentz‐type dielectric layer is placed between a graphene sheet and a 1DPC. (d) Schematic of TPA, in which the top layer is a non‐dispersive and lossless dielectric. Calculated LPA absorption spectra for TM (b) and TE (c) wave as a function of incident angle and frequency, and TPA absorption spectra for TM (e) and TE (f).

In LPA, the complex dielectric function $\varepsilon_{s}\left(\omega \right)$ for the top layer is modeled using the Lorentz permittivity function, expressed as [40].

(1) $$\varepsilon_{s}\left(\omega \right)=\varepsilon_{\infty }+\frac{f_{\text{osc}}\cdot \omega_{\text{TO}}^{2}}{\omega_{\text{TO}}^{2}-\omega^{2}-i\Gamma_{\text{TO}}\omega },$$

where $\varepsilon_{\infty }$ is the high‐frequency permittivity, $\omega_{\text{TO}}$ is the transverse optical phonon resonance frequency, $\Gamma_{\text{TO}}$ is the damping coefficient, $f_{\text{osc}}$ is the oscillator strength, and ω=2πf is the angular frequency. LPA use the following parameter values: $\varepsilon_{\infty }=5$, $\omega_{\text{TO}}=2\pi \times 1.76\times 10^{12}$ rad/s, $\Gamma_{\text{TO}}=2\pi \times 0.05\times 10^{12}$ rad/s, and $f_{\text{osc}}=1$.

A monolayer graphene sheet is placed at the air/top‐layer interface and treated as optically thin. Its electromagnetic response is described by a Drude‐like intraband conductivity expression suitable for the THz range [41, 42]:

(2) $$\sigma \left(\omega \right)=\frac{ie^{2}k_{B}T_{g}}{\pi \hbar^{2}\left(\omega +i/\tau \right)}\left[\frac{E_{F}}{k_{B}T_{g}}+2\;\ln\left(\exp\left(-\frac{E_{F}}{k_{B}T_{g}}\right)+1\right)\right],$$

where $T_{g}$ is the graphene temperature, $E_{F}$ is the Fermi energy, τ is the relaxation time, and $k_{B}$ is Boltzmann's constant. Here, the parameters are selected as: $T_{g}=300\;K,\;E_{F}=0.7\;eV,\tau =1\;ps,\;k_{B}=1.38\times 10^{-23}\;J/K$.

The 1DPC comprises alternating dielectric layers with refractive indices $n_{a}=1.5$ and $n_{b}=4$. The corresponding quarter‐wave thicknesses are given by $d_{a}=\lambda_{c}/\left(4n_{a}\right),d_{b}=\lambda_{c}/\left(4n_{b}\right)$, where the central design wavelength is $\lambda_{c}=172\;\mu m$. The center frequency is selected to match the Lorentz resonance and graphene Drude response while achieving phase and amplitude matching, thereby maximizing the absorption rate and ensuring the ability to work in wide‐angle ranges. The top layer is placed above the 1DPC and modeled using the Lorentz medium. The thickness of the top layer is defined as $d_{s}=\lambda_{c}/4\;ℜ\left(n_{s}\right)$.

The proposed structure is compatible with standard planar thin film fabrication processes [43]. The 1DPC can be prepared by sequential deposition of alternating dielectric layers with quarter‐wave optical thicknesses on a substrate, using conventional methods such as sputtering, evaporation, or atomic layer deposition, depending on the material system [17, 44]. The Lorentz dielectric top layer can then be deposited as a continuous film with thickness $d_{s}$. After that, a graphene monolayer can be introduced at the top layer interface through a conventional transfer process [45]. Since the present design does not rely on subwavelength lithography or patterned resonators, its fabrication route is simpler than that of many metasurface absorbers [46]. In practice, the main process requirements are accurate control of dielectric thickness and preservation of graphene quality both of them can be monitored through standard optical and electrical characterization methods [47, 48].

In addition, the polarization‐dependent absorption behaviors are considered under both TM and TE polarization, focusing on the comparison between TPA and LPA. In the TPA configuration, the top layer between the graphene sheet and the distributed Bragg reflector (DBR) is a lossless dielectric with a fixed refractive index of $n_{s}=1.9$. The DBR stack was constructed with alternating layers of $n_{a}=1.46$ and $n_{b}=1.9$ with T=20 periods, ensuring a well‐defined photonic bandgap supporting localized interfacial resonances. The thicknesses of each layer in the DBR followed a quarter‐wavelength condition, given by $d_{a}=\lambda_{c}/\left(4n_{a}\right)$ and $d_{b}=\lambda_{c}/\left(4n_{b}\right)$, where $\lambda_{c}=300\;\mu$m corresponds to the central design frequency, and the graphene conductivity followed a Drude model with parameters consistent with those mentioned before.

The Lorentz dielectric function $\varepsilon_{s}\left(\omega \right)$ is evaluated over a frequency range from 0.5 THz to 2 THz. The refractive index as $n_{s}=R\left[\sqrt{\varepsilon_{s}\left(\omega \right)}\right]$. Specifically, $n_{s}$ is selected using a frequency near 1.94 THz, which lies within the designed resonance band of the absorber. This choice ensures that the top layer thickness $d_{s}$ is computed where the real part of the permittivity is unchangeable, leading to accurate phase control and strong absorption performance across a wide angular range.

To analyze the multilayer system, transfer matrices specific to TM and TE polarizations were employed. For TM waves, the interface transfer matrix is given by [49].

(3) $$D_{p}=\frac{1}{2}\left(\begin{array}{cc} 1+\eta_{p}+\xi_{p} & 1-\eta_{p}-\xi_{p}\\ 1-\eta_{p}+\xi_{p} & 1+\eta_{p}-\xi_{p} \end{array}\right),$$

where $\eta_{p}=\varepsilon_{a}k_{lz}/\varepsilon_{l}k_{az},\xi_{p}=\sigma k_{lz}/\varepsilon_{0}\varepsilon_{l},$with $k_{az}=\omega /c\;\cos\;\theta$ and $k_{lz}=k_{0}\sqrt{\varepsilon_{l}-\varepsilon_{a}\sin^{2}\;\theta }$.

For TE polarization, the corresponding matrix is [49].

(4) $$D_{s}=\frac{1}{2}\left(\begin{array}{cc} 1+\eta_{s}+\xi_{s} & 1-\eta_{s}+\xi_{s}\\ 1-\eta_{s}-\xi_{s} & 1+\eta_{s}-\xi_{s} \end{array}\right),$$

where $\eta_{s}=k_{lz}/k_{az},\xi_{s}=\sigma \mu_{0}/k_{az}$ and $\mu_{0}$ is the vacuum permeability. These matrices control the coupling between the layers, fully accounting for the graphene dispersion properties of the top layer.

Figure 1b,c presents the angle and frequency dependent absorption spectra of the LPA for both TM and TE polarizations, whereas Figure 1e,f shows the corresponding results for the TPA. That shows that both TPA and LPA structures exhibit strong absorption performance over a wide angle range (0°–80°), while also demonstrating efficiency in achieving polarization‐insensitive absorption. The minimal performance difference between TM and TE modes highlights the general polarization robustness of both the LPA and TPA. Figure 1b,c show the angular evolution of the absorption spectra for the TM (0.95THz—1.22THz) and TE (0.95THz—1.18THz) polarizations, respectively. A clear redshift is observed in both cases with increasing incidence angle. In particular, the TPA structure exhibits a frequency shift exceeding 20% (Δf/f) under both polarizations, highlighting its susceptibility to angular dispersion. Figure 1e,f display the angular absorption characteristics under TM and TE polarizations, respectively. In Figure 1e for the TM mode, it maintains a high absorption rate of at least 92% within the range of 0°‐80°, and the resonant frequency ranges from 1.48THz at normal incidence to 1.57THz at the incident angle of 80°, corresponding to a relative frequency shift of approximately 5.7%. For the TE mode (Figure 1f), the same structure maintains PA within the incident angle range, and the displacement is relatively small. From 1.48THz to 1.53THz, the relative displacement is only 3.2%. Relative to TPA, the Lorentz dielectric's frequency‐dependent permittivity reduces the shift of the absorption peak with angle. Taken together, the results indicate that Lorentz dielectric improves dispersion control relative to TPA.

### 2.2 Phase‐Amplitude Matching Conditions and Angular Stability Mechanism of LPA

To describe and elucidate the mechanism of perfect THz absorption achieved by dispersion control in LPA structures, as well as analyze the similarities and differences in the working mechanisms of LPA and TPA, the physical processes behind it are next systematically analyzed using a phase‐amplitude matching framework. This approach indicates why and how the Lorentz‐type dielectric in LPA achieves wide‐angle and efficient absorption across varying incident angles. The matching condition of the electromagnetic field amplitudes at upper and lower interfaces of the top layer can be expressed as [50].

(5) $$\left(\begin{array}{c} 1\\ r_{\text{Gra}} \end{array}\right)\propto \left(\begin{array}{c} e^{i\Phi }r_{\text{DBR}}\\ e^{-i\Phi } \end{array}\right),$$

where $r_{\text{Gra}}$ and $r_{\text{DBR}}$ are the reflection coefficients at graphene‐top layer and top layer‐1DPC interfaces, respectively, and Φ represents the phase delay across the top layer.

From the intrinsic field relationship in Equation (5) and enforcing the continuity of the tangential fields, the cavity supports a nonradiative resonance only when both the amplitude and phase constraints are satisfied. To ensure resonance across the graphene‐top layer‐1DPC interface, phase and amplitude matching condition must be satisfied, as derived from the intrinsic field continuity relations in Equation (4). An amplitude condition that ensures top layer reflection and a phase condition that requires constructive resonance need to be satisfied. The amplitude requirement is

(6) $$|r_{\text{Gra}}|\approx |r_{\text{Gra}}\;r_{\text{DBR}}\;e^{2i\Phi }|,$$

to ensure resonance at the top layer. Simultaneously, the phase condition requires the total phase contribution to satisfy

(7) $$\arg\left(r_{\text{Gra}}\;r_{\text{DBR}}\;e^{2i\Phi }\right)\approx 0,$$

Figure 2a depicts the spectral evolution of the total phase $\arg\left(r_{\text{Gra}}r_{\text{DBR}}e^{2i\Phi }\right)$ under normal incidence (θ=0°) for both TPA and LPA configurations. Φ represents the accumulated phase at the top layer and is used to analyze the phase matching condition in the LPA and TPA structures. $\Phi_{\text{TPA}}$ curve (blue line) crosses zero near 0.95 THz, marked by a black dot. $\Phi_{\text{LPA}}$ curve (red dashed line) satisfies the phase‐matching condition near 1.5 THz. These two frequencies correspond to the PA points observed in Figure 1b,d at 0°, respectively, indicating the fulfillment of the excitation condition described by Equation (7). Complementarily, Figure 2b presents the corresponding amplitude behavior under normal incidence of LPA. Near 1.5 THz, amplitude terms $\left|r_{\text{Gra}}\right|$ and $\left|r_{\text{Gra}}r_{\text{DBR}}e^{2i\Phi }\right|$ exhibit close alignment, confirming that the amplitude condition defined by Equation (6) is simultaneously satisfied. These results confirm that the phase and amplitude conditions required are simultaneously met near 1.5 THz. Notably, this resonant frequency corresponds precisely to the PA observed in Figure 1e, further validating that the phase and amplitude matching conditions (Equations 6 and 7), which can accurately describe the PA characteristic in LPA.

**FIGURE 2:**
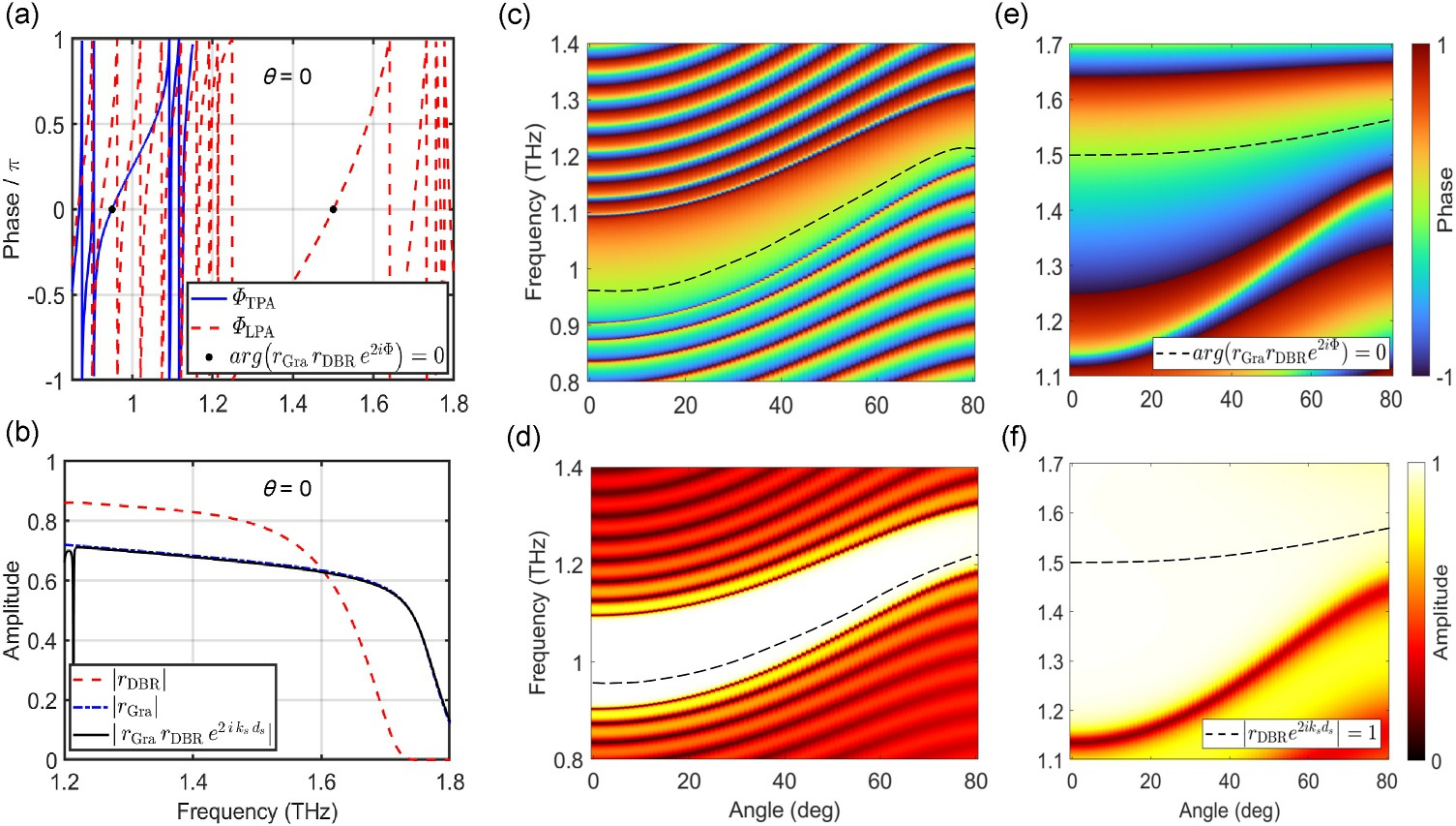
(a) Φ at normal incidence (θ=0°) for TPA (blue) and LPA (red) structures. Black dots indicate frequencies where the phase condition $\arg\left(r_{\text{Gra}}\;r_{\text{DBR}}\;e^{2i\Phi }\right)=0$ is satisfied, corresponding to PA frequencies seen in Figure 1b,d. (b) Amplitude response of TPA and LPA at θ=0°. At resonance, the curves for $\left|r_{\text{Gra}}\right|$ and $\left|r_{\text{Gra}}\;r_{\text{DBR}}\;e^{2i\Phi }\right|$ coincide, satisfying the amplitude matching criterion. (c) Phase evolution of the TPA structure as a function of incidence angle and frequency; the dashed black line indicates the fulfillment of the phase resonance condition. (d) Amplitude excitation map for the TPA structure, showing the angular dependence of the matching condition; the dashed line marks resonance. (e) Same as (c), but for the LPA structure, exhibiting superior phase stability. (f) Corresponding amplitude excitation map for LPA, demonstrating improved matching and reduced angular sensitivity.

Figure 2c,d presents the evolution of phase and amplitude characteristics for the TPA configuration under varying incidence angles. In Figure 2c, the total phase $\arg\left(r_{\text{Gra}}r_{\text{DBR}}e^{2i\Phi }\right)$ shows a significant angular shift near the resonant frequency, indicating that although the phase matching condition is satisfied, its spectral position varies with the incidence angle. The track of PA, highlighted by the black dashed line, aligns with this phase evolution, confirming that the observed angular dispersion in the absorption spectrum corresponds to a gradual offset of the matching condition from its original zero‐phase point. This behavior is consistent with the absorption blueshift shown previously in Figure 1b,c, where the resonance frequency undergoes a relative shift exceeding 20% as the incident angle increases from 0° to 80°. Figure 2d further supports this observation by depicting the amplitude response. Here, the simplified amplitude condition, $|r_{\text{DBR}}e^{2i\Phi }|\approx 1$, reveals a frequency shift in the region where this criterion is satisfied, again mirroring the drift in PA seen in the angular absorption spectra. The variation with angle suggests that the non‐dispersive TPA structure is sensitive to angular shifts in the resonance condition. Although PA is still observed, its spectral position exhibits sensitivity to the incidence angle, indicating limited control over angular dispersion.

In contrast, Figure 2e,f illustrates the phase and amplitude behavior of the LPA structure. As seen in Figure 2e, the phase‐matching contour remains nearly flat and localized near 1.5 THz across the full range of incidence angles (0°–80°), demonstrating strong angular stability. This behavior stems from the Lorentzian dielectric's frequency‐dependent permittivity, which effectively compensates for angle‐induced changes and maintains the phase condition described by Equation (8). Similarly, Figure 2f shows that the amplitude‐matching condition, simplified to $|r_{\text{DBR}}e^{2i\Phi }|\approx 1$, holds consistently along the positions of PA indicated by the black dashed line. This consistent match across the angular span confirms that the amplitude constraint in Equation (6) is also necessary for the LPA configuration. These findings validate that both phase and amplitude excitation criteria are robustly met across a wide angular range in the dispersive structure, supporting the enhanced angular insensitivity observed in Figure 1d. The ability of the Lorentz‐type dielectric to stabilize both phase and amplitude matching condition support the superior performance of LPA over TPA designs.

To explain the wide‐angle PA mechanism of the LPA, we examine how the total phase Φ accumulated in the Lorentz top layer responds to changes in the incidence angle θ. In TPA structures, the top layer is non‐dispersive, meaning its refractive index remains constant over frequency. As the incident angle increases, the effective phase accumulation distance along the normal direction of the layer becomes shorter. This leads to a decrease in the phase accumulated across top layer. Since the resonance condition depends on satisfying Equation (7), this reduction shifts the resonance to a higher frequency.

When the incident angle increases, in order to maintain the frequency of PA unchanged, materials with appropriate dielectric properties are required. Lorentz‐type media satisfy this requirement due to their frequency‐dependent permittivity. The real part of the dielectric constant $R\varepsilon_{s}\left(\omega \right)$ exhibits dispersive characteristics: it increases with frequency in the region below the resonance and decreases above. Therefore, when LPA operates below the resonance frequency, where $\partial R\varepsilon_{s}/\partial \omega >0$. This dispersion can provide a compensatory mechanism. This compensates for the reduced propagation depth and restores the accumulated phase. In this way, the system compensates the angular‐induced phase loss, maintains a non‐dispersion phase matching, and enables PA to persist even at oblique angles.

To further understand this phase compensation from a mathematical perspective, we can consider the following approach. Below the resonance frequency $\omega_{0}$, $R\varepsilon_{s}\left(\omega \right)$ has a positive slope $\left(\partial R\varepsilon_{s}/\partial \omega >0\right)$, enabling two opposing effects: (1) a decrease in Φ resulting from the angular increase of the phase accumulation term cosθ in Equations (8) and (2); and (2) an increase of $n_{s}$ via dispersion. These effects compensate each other and enable wide‐angle PA. The total phase can be expressed as a function of both incidence angle and the frequency‐dependent permittivity:

(8) $$\Phi \left(\theta ,\omega ,\varepsilon_{s}\right)=\frac{\omega }{c}\cdot n_{s}\left(\omega \right)\cdot d_{s}\cdot \cos\;\theta .$$

This expression highlights how the phase accumulation Φ is jointly influenced by two parameters that change with incidence angle: the angular projection factor cosθ and the refractive index $n_{s}\left(\omega \right)$. As θ increases, cosθ leads to a reduction in the total accumulated phase Φ according to Equation (8). However, the change in θ also shifts the resonance frequency ω(θ). Due to the dispersive nature of the Lorentz medium, this frequency shift leads to an increase in $R\left[\varepsilon_{s}\left(\omega \right)\right]$, thereby increasing $n_{s}\left(\omega \right)$. Because $n_{s}\left(\omega \right)$ increases as ω shifts upward near resonance, it partially compensates the decrease in cosθ. This results in a relatively small net change in Φ with increasing angle, maintaining the phase‐matching condition required for PA at 0°—80°. In contrast, in a TPA system with a nondispersive dielectric, $n_{s}$ is fixed and does not compensate for the angular variation in cosθ. As a result, the only way to maintain a constant Φ in TPA is to introduce a shift in ω to adapt to the angular changes. Unlike TPA, which requires frequency shifting to maintain a constant Φ due to its fixed refractive index, LPA utilizes the dispersion of the top layer medium to compensate for angular dispersion, thereby achieving PA over a wide angle without dispersion and without the need to adjust frequency or structural parameters.

To quantify the compensation effect in Equation (8), we further introduce a phase drift measure and the corresponding phase compensation efficiency. We define the phase drift for each structure as

(9) $$\Delta \phi_{X}\left(\theta \right)=\phi_{X}\left(\theta \right)-\phi_{X}\left(0\right),$$

where

(10) $$\phi_{X}\left(\theta \right)=\arg\;\left[r_{\text{Gra},X}\left(\theta ,f_{0}\right)\;r_{\text{DBR},X}\left(\theta ,f_{0}\right)\;e^{2i\Phi_{X}\left(\theta ,f_{0}\right)}\right],X\in \left\{TPA,LPA\right\},$$

and $f_{0}$ is chosen as the normal incidence resonance frequency of the absorption channel under discussion. On this basis, the phase‐compensation efficiency is defined as

(11) $$\eta_{\phi }\left(\theta \right)=1-\frac{\left|\Delta \phi_{\text{LPA}}\left(\theta \right)\right|}{\left|\Delta \phi_{\text{TPA}}\left(\theta \right)\right|}.$$

Figure 3 presents the corresponding quantitative results. The phase drift of the LPA remains consistently smaller than that of the TPA over the full angular range, as shown in Figure 3a. In Figure 3b the corresponding $\eta_{\phi }\left(\theta \right)$ stays at a high positive level, which indicates that the LPA structure suppresses most of the angle phase variation relative to TPA structure. These results provide direct quantitative support for the reduced angular drift observed in the absorption spectra and further confirm that the Lorentz dielectric improves phase stability under oblique incidence.

**FIGURE 3:**
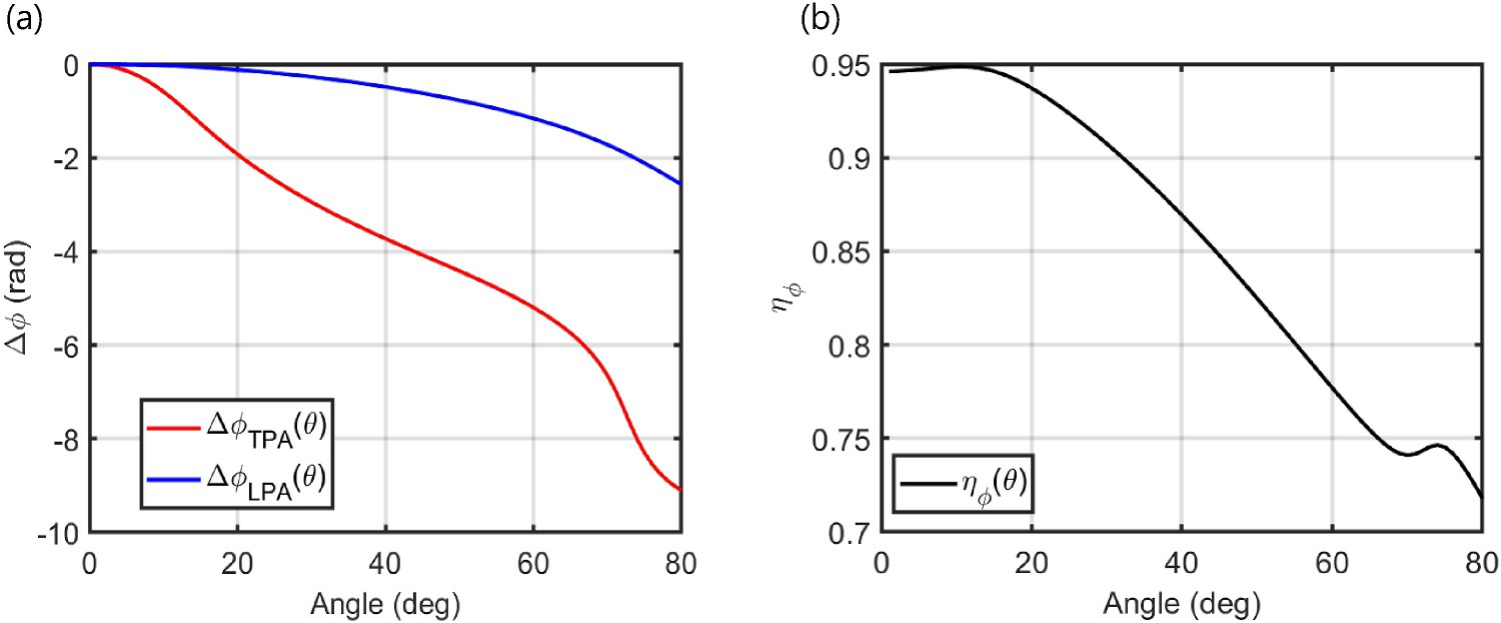
Quantitative evaluation of the phase compensation effect. (a) Angle phase drift $\Delta \phi_{\text{TPA}}\left(\theta \right)$ and $\Delta \phi_{\text{LPA}}\left(\theta \right)$ evaluated at the same reference frequency $f_{0}$. (b) Phase compensation efficiency $\eta_{\phi }\left(\theta \right)$ as a function of incident angle.

Furthermore, the phenomenon of PA in LPA and its matching conditions can be understood from the Lorentz model itself. In the Lorentz model, there are three key parameters, which are: the oscillator strength $f_{\text{osc}}$, the damping coefficient $\Gamma_{\text{TO}}$, and the offset $\Delta \omega =\omega_{\text{TO}}-\omega_{0}$ between the material resonance $\omega_{\text{TO}}$ and the uncoupled cavity‐mode frequency $\omega_{0}$. An increase in $f_{\text{osc}}$ enhances the magnitude of the imaginary permittivity near resonance, enhancing absorption strength and confining the resonance more tightly near the target frequency. This increases the absorption amplitude while reducing unnecessary secondary resonance. The damping rate $\Gamma_{\text{TO}}$ controls the spectral width of the resonance. A moderate value introduces sufficient broadband loss to mitigate angular drift without broadening the absorption band. The detuning Δω determines the spectral separation between the material resonance and the intrinsic cavity mode. Δω>0 ensures that the material resonance lies above the bare cavity mode, avoiding spectral overlap. This configuration shifts the PA frequency into a region where the Lorentz‐type dielectric function varies slowly, unwanted mode interactions are suppressed and dispersion is flattened, thereby limiting frequency shifts at oblique angles. In addition, temperature can also affect the Lorentz dielectric response. In a Lorentz‐type medium, thermal variation can modify both the real and imaginary parts of the permittivity through changes in the Lorentz parameters, and therefore alter the phase response, resonance position, and loss characteristics of the dielectric [51, 52]. In phonon Lorentz media, increasing temperature is commonly accompanied by a shift of the resonance frequency toward lower frequency and an increase in the damping parameter, whereas the strength parameter remains nearly unchanged [52]. As a result, the dispersive region of the dielectric function shifts and the loss linewidth broadens. These changes can modify the phase‐compensation condition, change the absorber bandwidth, and reduce the stability of the resonance under temperature variation.

To clarify how the Lorentz medium regulates angular stability, Figure 4 shows representative cases obtained by varying the resonance frequency $\omega_{\text{TO}}$ and the damping coefficient $\Gamma_{\text{TO}}$, while keeping the remaining parameters unchanged relative to the reference case in Figure 1e.

**FIGURE 4:**
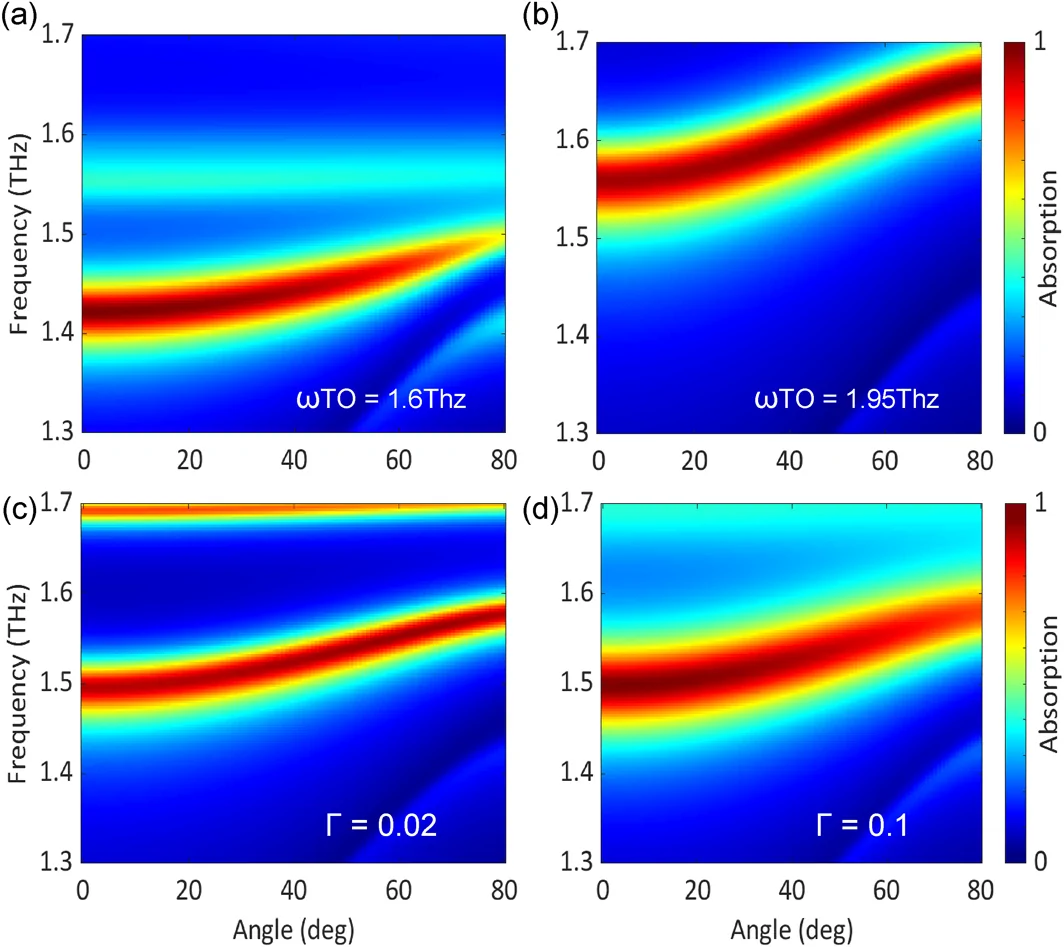
Angle resolved absorption spectra of the LPA for representative Lorentz parameter variations. (a) and (b) show the effect of changing the resonance frequency to $\omega_{\text{TO}}/2\pi =1.6$ THz and 1.95 THz, respectively. (c) and (d) show the effect of changing the damping coefficient to $\Gamma_{\text{TO}}/2\pi =0.02$ THz and 0.1 THz, respectively. All other parameters are kept the same as in the reference case.

Figure 4 shows that the angular response of the LPA is influenced by the Lorentz parameters, although the detailed trend is not governed by a single rule because the final response is determined by the combined effect of dielectric dispersion, cavity coupling, and phase‐amplitude matching. Compared with the reference case in Figures 1e and 4a shows that reducing $\omega_{\text{TO}}$ to 1.6 THz shifts the absorption band to a lower frequency and weakens the high angle perfect absorption condition. This result indicates that when the material resonance moves closer to the absorption frequency, the dielectric dispersion in that frequency range increases, so the phase matching compensation is no longer maintained over the full angular range. Figure 4b shows that increasing $\omega_{\text{TO}}$ to 1.95 THz shifts the absorption band to a higher frequency and leads to a more visible angular drift. In this case, the absorption band moves farther from the frequency range where the Lorentz response provides compensation, so the phase correction is reduced and the peak frequency changes more clearly with angle. Figure 4c,d show the effect of the damping coefficient. When $\Gamma_{\text{TO}}/2\pi$ is reduced to 0.02 THz in Figure 4c, the absorption band becomes narrower and the angular drift is reduced. This result shows that a smaller damping confines the Lorentz response to a narrower frequency interval, so the phase compensation is concentrated near the target channel. By contrast, when $\Gamma_{\text{TO}}/2\pi$ is increased to 0.1 THz in Figure 4d, the absorption band becomes broader and the perfect absorption condition is no longer maintained as clearly as in the reference case. The larger damping broadens the dielectric response and reduces the local phase, so the compensation becomes less precise and the resonance loses spectral sharpness. We choose $\omega_{\text{TO}}$ and $\Gamma_{\text{TO}}$ because these two parameters directly determine the position and width of the Lorentz response. These representative cases clarify the main trends by which the Lorentz parameters regulate angular stability in the present design, although the complete response is still determined by the combined effect of resonance position, damping, dielectric dispersion, cavity coupling, and phase‐amplitude matching.

In the proposed LPA structure, resonant absorption is governed by electric‐field redistribution near the graphene/Lorentz interface and within the dispersive top layer. At resonance, the electric field is concentrated near the graphene sheet, where graphene contributes conductive loss, whereas the Lorentz dielectric provides a frequency‐dependent phase delay and controlled loss [53]. As a result, the cavity stores more electromagnetic energy in the regions where dissipation occurs, which reduces the reflected power and enhances the absorption efficiency [5, 54]. In LPA, graphene serves as the current carrying boundary, and the Lorentz dielectric shapes the local field, phase accumulation, and loss channel required for PA [55].

The electric‐field distribution also evolves with the incident angle, and this evolution determines the angular dispersion of the absorber [56]. As the angle increases, the in plane wave vector and the phase accumulated in the cavity are modified, so the electric field becomes redistributed across the structure [57]. When the Lorentz dielectric continues to provide sufficient phase compensation, the field maximum remains concentrated near the top layer and the absorption peak exhibits only a small spectral shift [56, 58]. When the field localization near the top layer becomes weaker, the resonance condition moves and the angular dispersion becomes more pronounced [56, 58]. Accordingly, the wide‐angle response of the proposed structure originates from the ability of the Lorentz dielectric to preserve the interfacial field confinement ability and the phase‐amplitude matching condition over a broad range of incident angles.

On the basis of the results and analysis, it can be concluded that the fundamental difference between TPA and LPA is that the TPA top layer structure exhibits spectral drift because of its non‐dispersive dielectric properties and the LPA top layer structure leverages Lorentz‐type dispersion to stabilize the resonance conditions. Unlike TPA designs that rely on geometrical tuning, resonator arrays, or complex nanostructuring to achieve wide‐angle operation [59, 60], the LPA achieves this performance by engineering the top layer's permittivity spectrum. The use of a Lorentz‐type dispersion not only enhances confinement and reduces angular dispersion but also simplifies the physical layout, avoiding added structural complexity. Furthermore, the absorption peaks predicted according to the phase and amplitude matching conditions in Equations (6) and (7) are in high agreement with the results of the full‐wave simulations, suggesting that the absorption properties of the LPA structure can be explained by analytical theory. This highlights the reliability of the proposed mechanism. Compared with earlier methods that rely on strong coupling or hybrid plasmonic excitonic interactions and often demand tight fabrication tolerances with limited tunability [61, 62], we realize PA by enforcing phase and amplitude matching conditions. In LPA, the Lorentz dielectric supplies a frequency‐dependent phase response that offsets angle induced phase shifts while maintaining the amplitude condition over a wider incidence range. As a result, LPA shows reduced angular dispersion within a wide angle range while preserving subwavelength thickness and avoiding additional fabrication steps; this contrasts with TPA and offers a useful approach for PA applications.

### 2.3 Geometric Tunability of Lorentz Perfect Absorption via Top‐Layer Thickness and Photonic Bandgap Engineering

It is also important to fully understand and grasp the tunability of the LPA based on a full understanding of its working mechanism. Figure 5 shows the angle absorption spectra of LPA structures for different geometric configurations. Without losing generality, we take the analysis of the THz absorption characteristics of the TM mode as an example here. Then, we examine the effect of varying the top layer thickness $d_{s}$, which was previously fixed at 45.6μm. The top‐layer thickness is set to 35μm, 45.6μm, and 55μm to assess its influence of PA. For $d_{s}=55\;\mu m$, the maximum absorption within 0°–80° reaches about 98%, and the absorption at 80° remains around 90%. For $d_{s}=35\;\mu m$, the maximum absorption reaches about 95%, whereas the absorption at 80° decreases to about 80%. As shown in Figure 5a, increasing the top‐layer thickness leads to a clear redshift of the absorption peak. This behavior is attributed to the phase‐matching condition required for PA at the interface. A thicker Lorentz layer delays phase accumulation across the interface, which shifts the resonance to lower frequencies. Varying the top‐layer thickness changes the round‐trip phase. We choose the thickness so that Equation (8) remains satisfied at the target frequency over the designed angle range. As shown in Figure 5b, decreasing the top‐layer thickness leads to a blueshift of the absorption peak. As the incident angle increases, the PA condition is no longer maintained over the full angular range. This indicates that the spectral tunability introduced by $d_{s}$ is not independent, but is limited by the phase‐amplitude matching condition. Within a suitable thickness range, the structure still supports PA. However, when the thickness variation exceeds this range, the matching condition becomes insufficient to compensate the angle‐dependent phase variation, resulting in degraded absorption at large incident angles. Hence, $d_{s}$ determines not only the resonance frequency but also the allowable thickness range for sustaining wide angle PA. This thickness‐dependent behavior shows that the LPA provides spectral tunability while retaining a certain fabrication tolerance for wide‐angle absorption.

**FIGURE 5:**
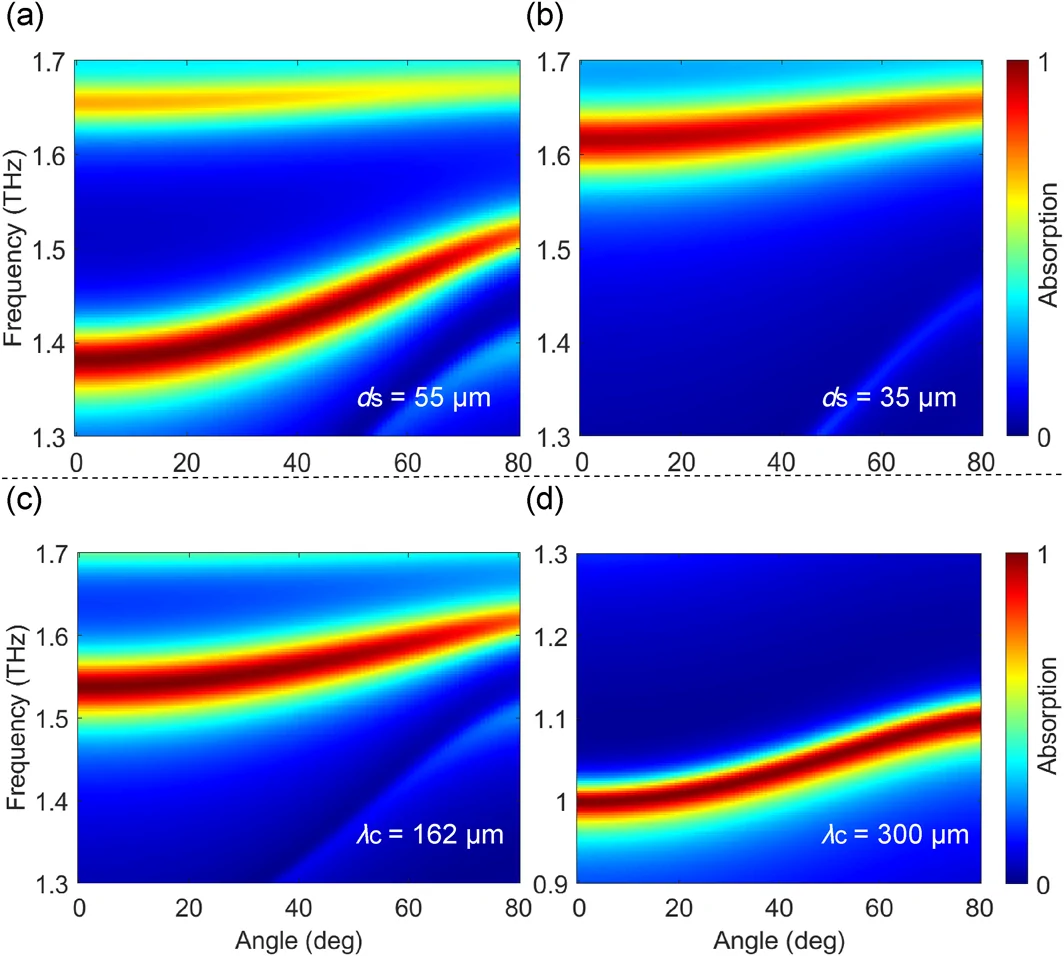
Angle‐resolved absorption spectra under TM polarization for different top‐layer configurations. (a, b) The thickness of the Lorentz‐type top layer is varied with $d_{s}=55\;\mu m$ and 35μm, respectively. (c, d) The center wavelength $\lambda_{c}$ of the DBR is set to 162 μm and 300 μm, respectively.

We investigate the effect of modifying the center design wavelength $\lambda_{c}$, which defines the spectral position of the photonic band gap (PBG) through the Bragg layer thickness relations $d_{a}=\lambda_{c}/\left(4n_{a}\right)$ and $d_{b}=\lambda_{c}/\left(4n_{b}\right)$. The selected values were 162μm, 172μm and 300μm, and the Lorentz top‐layer thickness was fixed at $d_{s}=\lambda_{c}/\left(4Rn_{s}\right)$. As shown in Figure 5c,d, increasing $\lambda_{c}$ causes the absorption peak to shift to lower frequencies. This shift occurs because a larger $\lambda_{c}$ results in thicker DBR layers, which in turn reduces the Bragg reflection frequency and shifts the PBG downward. The resonant state, confined within this bandgap, follows this spectral shift. Adjusting the 1DPC center wavelength sets the reflection phase and magnitude. We align these with the top‐layer phase so that Equation (8) remains satisfied over angle while amplitude balance is retained. The wide‐angle absorption performance remains consistently stable, indicating that spectral tunability and angular insensitivity can be achieved simultaneously by leveraging the Lorentz medium, confirming the flexibility of the graphene‐Lorentz‐DBR system. By independently tuning either the top‐layer thickness or the center frequency of the DBR structure, the resonance frequency can be manipulated without compromising angular robustness. This tunability provides a practical strategy for designing multifunctional THz absorbers with a high spectral selectivity and wide‐angle operability.

In contrast, TPA designs fix the resonance frequency through static geometry and nondispersive dielectric materials [63]. Although previous studies have demonstrated spectral tunability in THz absorbers through geometric reconfiguration or materials with tailored dispersion, such approaches often require multilayer stacking, complex metamaterial patterning, or external biasing to achieve the desired outcomes [64, 65, 66]. For example, structures employing resonator arrays or hybrid metal‐dielectric designs usually rely on local resonances and geometric scaling laws to tune absorption bands. However, these methods often lead to narrowband responses or increased angular sensitivity due to limited phase control [67]. Additionally, graphene‐based absorbers in recent literature exploit tunable chemical potential or external voltage to shift the absorption peak, but such tuning is limited in range and often degrades with angle of incidence [68].

By leveraging the dispersive properties of the Lorentz‐type dielectric, the proposed LPA structure offers a compact, easy fabrication solution that maintains wide angle absorption while enabling spectral tunability. This approach sets the LPA apart from both TPA and other tunable absorbers schemes by achieving frequency tunability which realizes angular robustness and reduces structural complexity.

### 2.4 DBR Contrast and Period Effects on Angular Absorption Performance

In addition to the two parameters mentioned in Figure 5, the influence of DBR characteristics on LPA THz absorption characteristics was also discovered. Figure 6 examines the influence of DBR refractive index contrast and the number of Bragg periods of absorption. Across all cases, the Lorentz‐type dielectric and graphene interface are preserved to isolate the effect of modifying the DBR stack.

**FIGURE 6:**
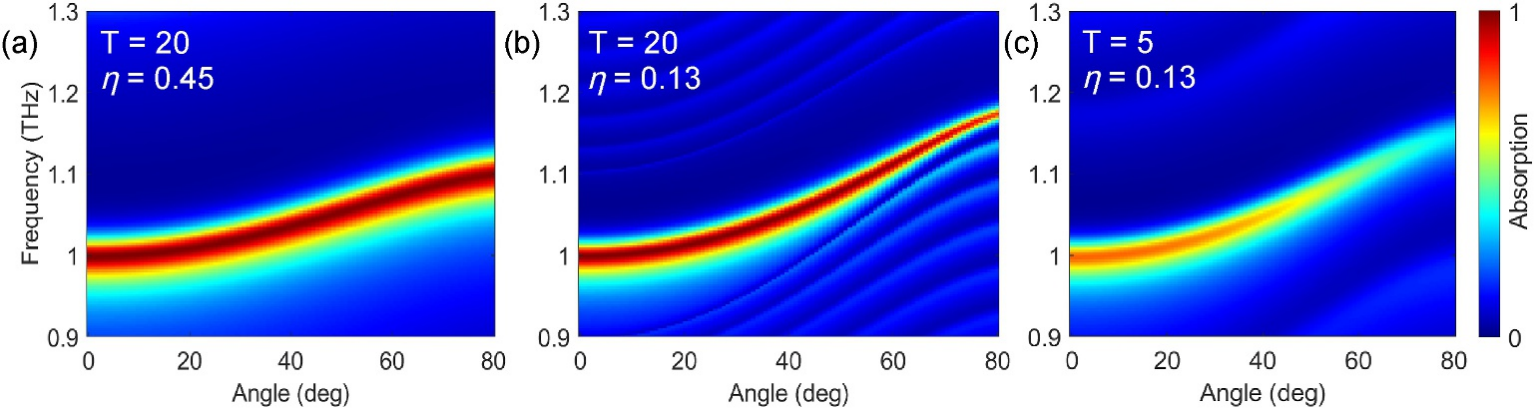
Angle‐resolved absorption spectra for LPA structures under TM polarization with different DBR configurations. In all cases, the Lorentz‐type top layer and graphene interface are preserved. Panel (a) corresponds to a DBR with refractive indices $n_{a}=1.5$, $n_{b}=4.0$, T=20, η=0.45; panel (b) uses a DBR with $n_{a}=1.46$, $n_{b}=1.9$, T=20, η=0.13; and panel (c) shows the response for a DBR with T=20 and the same η indices as in (b).

Figure 6a displays the absorption for a DBR structure with a high refractive index contrast ($n_{a}=1.5$, $n_{b}=4.0$) and 20 alternating periods. The DBR contrast ratio as $\eta =\frac{n_{b}-n_{a}}{n_{b}+n_{a}}$. This configuration yields a spectrally narrow angularly PA band centered around 1.0 THz, exhibiting minimal dispersion over the full range of incidence angles (0°–80°). The strong Bragg reflection afforded by the high index contrast ensures a wide photonic bandgap and efficient optical feedback, supporting the phase and amplitude matching conditions necessary. This result demonstrates that with sufficiently high contrast, the system achieves robust absorption without relying on a large number of Bragg cycles. In contrast, Figure 6b corresponds to the LPA with a lower DBR contrast ($n_{a}=1.46$, $n_{b}=1.9$) with the same number of periods (20). Although it still supports broad‐angle absorption, the spectral contour becomes slightly more sensitive to angle, and small oscillatory features emerge at higher incidence. These stem from weaker reflectivity and partial transmission through the DBR, causing Fabry‐Pérot‐like leakage that indicate a decline of the resonance condition stability. Figure 6c further degrades the DBR by reducing the number of periods to only five while maintaining the low‐contrast index pair. In this case, the absorption performance decreases and exhibits angle and spectral broadening. This reduction arises because the low contrast and Bragg layers cannot sustain the resonance and phase accumulation needed for achieving strong field confinement and near‐unity absorption.

Unlike the high‐contrast structure shown in Figure 6a, this configuration maintains sufficient optical feedback even with fewer layers. To quantify these effects. For the high‐contrast design in Figure 6a, η=0.45, whereas the low‐contrast configuration in Figure 6b,c has η=0.13. High contrast DBRs generate a broader and deeper photonic bandgap, enhancing reflectivity and field confinement near the interface. When the contrast ratio η is high, strong back‐reflection is maintained over a wide angular range, allowing the absorption band to remain well‐confined and efficient (Figure 6a). In contrast, low η DBRs yield weaker feedback and allow energy leakage at oblique angles, reducing both confinement and absorption strength (Figure 6c). Thus, a high η design ensures angular dispersion control and strong absorption. The results suggest that achieving a contrast ratio of at least η≥0.4 is essential to realize angularly robust and spectrally selective absorption, particularly when the number of DBR cycles is minimized.

Another important advantage of the LPA is that it has fewer DBRs. Mechanistically, the Lorentz dielectric provides both the required loss and a frequency‐dependent response that maintains the cavity condition near the target frequency. This reduces the dependence on very high reflector magnitude, so a smaller number of DBR periods suffices, whereas the TPA case typically needs more periods to reach the same absorption. Whereas TPA often rely on 8–10 periods to ensure adequate reflectivity and optical confinement, the model achieves a comparable PA performance with only 5 periods which achieve the same functionality with less complexity. This reduction in periods simplifies the manufacturing process and lowers material costs, while also making the device more compact. This approach extends PA to miniature THz photonic and flexible optoelectronic systems under tight space and fabrication constraints. Similar results have been demonstrated in recent studies [69, 70], but these approaches typically depend on more intricate methods, such as metamaterial coatings or complex nanostructure patterning, to reduce the DBR periods [27, 71]. In contrast, LPA design preserves the structural simplicity and planar symmetry, relying solely on resonant dispersion control via a Lorentzian medium. In summary, the results shown in Figure 4 indicate that high DBR contrast is important in achieving fewer Bragg periods while maintaining effective absorption. This provides a design solution for constructing compact LPA structures without angular dispersion performance.

### 2.5 Multi‐Channel Absorption Enabled by Multiple Lorentz Resonances

In many important applications, multi‐channel PA properties are required. In previous studies, graphene Tamm structures have been used to achieve THz multi‐channel PA [72]. In this paper, we achieve multichannel THz PA by applying the phase‐amplitude matching mechanism in LPA, resulting in reduced angular dispersion within the designed range and structural simplicity. To further expand the capabilities of the structure, we explored multi‐channel PA by modifying only the top layer while keeping both the monolayer graphene and DBR unchanged. This top layer was designed using a Lorentz‐type dielectric with a high‐frequency permittivity $\varepsilon_{\infty }=4$ and five distinct resonance frequencies: 2.5, 2.0, 1.65, 1.1, and 0.5 THz. The TE mode was used. The thickness of the top layer was set to $d_{l}=8\;\mu m$ which ensured the amplitude and phase matching. Although the absorption peaks correspond to different Lorentz resonance frequencies, PA at these frequencies is achieved with the same top layer thickness. This occurs because the phase matching condition in the LPA is not solely determined by the thickness of the top layer but also by the frequency‐dependent permittivity of the Lorentz medium. As the real part of the dielectric function $R\varepsilon_{s}\left(\omega \right)$ varies with frequency, the effective refractive index and its spectral slope contribute to the phase response required for matching. This characteristic distinguishes LPA from TPA and allows for multi‐frequency PA under a unified top layer thickness. Introducing multiple Lorentz resonances into the dielectric response creates distinct spectral regions that support phase and amplitude matching conditions, resulting in enhanced field localization and the emergence of multiple absorption peaks.

Under normal incidence, the absorption spectrum shown in Figure 7a shows three resonant peaks exceeding 99%, confirming the successful excitation of multiple high efficiency absorption channels. As shown in Figure 7b, the PA regions remain non‐dispersive across the angular range from 0° to 80°. This angular robustness indicates that the matching conditions for both the amplitude and phase are preserved despite changes in the incidence angle. This is because of the gradual change in the Lorentz‐type dispersion, which alleviates the phase mismatch usually introduced by oblique incidence. The robustness of this behavior is further demonstrated in Figure 7c, where the angle‐absorption spectra at 0°, 20°, 40°, 60°, and 80° show that all three resonant channels maintain PA levels with minimal spectral drift. The relative deviation of the resonance position, quantified by Δf/f, is approximately 5.2% at the most, and the angular dispersion control provided by the Lorentz top layer is verified. It offers tolerance to angle variation owing to its field orientation being parallel to the interface and perpendicular to the incidence plane, resulting in minimal impedance changes with the angle.

**FIGURE 7:**
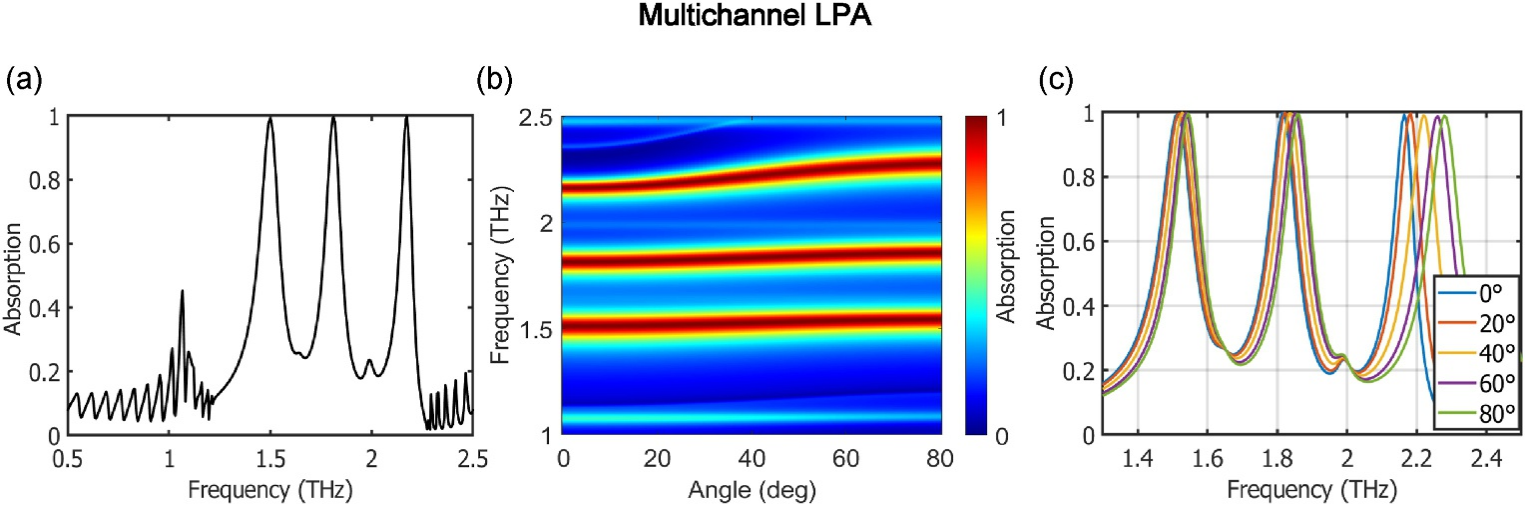
(a) Absorption spectrum at normal incidence designed using multiple Lorentz resonances to achieve multichannel PA. (b) The absorption performance of the structure under varying incident angles ranging from 0° to 80°. (c) The absorption spectra plotted at different angles.

These findings confirm that using a compact Lorentz‐type dispersion can not only enable multiple discrete absorption bands but also suppress angular dispersion through stable resonance conditions across a wide incidence angles. Additionally, Lorentzian top layer(∼λ/22) is thinner and achieves a comparable absorption performance to recent multichannel perfect absorber designs that use hyperbolic metamaterials [27, 70]. In TPA structures, the thickness of the top layer is usually several times or even dozens of times that of LPA [73]. However, the LPA system achieves angle‐insensitive high‐efficiency multiband absorption without relying on complex geometrical structures or anisotropic materials. Previous studies have shown that achieving angularly robust multichannel absorption typically requires spatial arrangement of resonators or the use of thick lossy media [62, 74]. In contrast, Lorentz‐type dielectric top layer can realize tunable multiband performance. Moreover, although recent PA demonstrate broadband or multiband characteristics, they frequently lack theoretical predictability or are sensitive to angular variation due to their reliance on localized surface modes [75, 76]. LPA forms multiple channels by meeting the same phase‐amplitude matching conditions at several frequencies through Lorentz dispersion with a single thickness, whereas TPA relies on distinct geometric resonances, which raises structural complexity and yields larger angular dispersion between channels. In this context, the LPA mechanism presented here exhibits not only improved structural simplicity and polarization insensitivity but also provides physical interpretation through amplitude and phase matching theory. This makes the design particularly suitable for THz applications requiring spectral selectivity and angular tolerance, such as tunable filters, sensing platforms, and photonic cloaking devices.

Table 1 compares the proposed absorber with representative terahertz absorbers reported in the paper in terms of structure, channel number, incident‐angle range, absorption at the maximum reported angle, polarization response, structural thickness, and fabrication complexity. In recent years, several reported absorbers rely on patterned metasurfaces or composite active materials to obtain high absorption and angular tolerance. These approaches can provide good performance, but they also increase structural complexity. In contrast, the proposed graphene–Lorentz–DBR structure remains planar and avoids subwavelength patterning, which makes the design simpler from a fabrication perspective.

**TABLE 1:** Compare the proposed absorber with the terahertz absorbers reported in the paper.

Reference	Structure	Channels	Incident angle	Absorption at max angle	Polarization	Structural thickness	Fabrication
[25]	${\text{MoS}}_{2}$ + graphene + DBR	Multi	0°–40°	PA at 40°	TE/TM	1158.78 μm	Simple
[77]	Graphene + PTFE + Au	Single	0°–60°	>99% at 60°	TE/TM	14.7 μm	Difficult
[18]	Metasurface + PI + Al	Multi	0°–55°	>96% at 55°	TE	50.4 μm	Difficult
[78]	Graphene + ${\text{VO}}_{2}$ + Topas + Au	Multi	0°–50°	—	TE/TM	9.1 μm	Difficult
[27]	Graphene + spacer + PhC	Multi	0°	—	TE/TM	2050 μm	Simple
[79]	Graphene+ ${\text{VO}}_{2}$ + TOPAS + Au	Multi	0°–70°	>90% at 70°	TE/TM	5.82 μm	Difficult
This work	Graphene + Lorentz + DBR	Multi	0°–80°	≥92% at 80°	TE/TM	242.7 μm	Simple

From the absorption perspective, the proposed structure shows a clear advantage at large incident angles. The absorption remains higher than 92% at 80°, which extends the angular range beyond most of the compared studies. This result indicates that the Lorentz dielectric layer helps maintain the resonance condition under oblique incidence. Therefore, the proposed absorber provides a useful balance among wide‐angle absorption, multichannel operation, TE/TM compatibility, and planar fabrication simplicity.

In this work, we propose a new approach by integrating a Lorentz‐type dispersive dielectric layer into a Tamm absorber framework constructed from a 1DPC and a graphene interface. This structure addresses several persistent challenges associated with conventional Tamm designs, including angular sensitivity, limited spectral tunability, and the need for relatively thick dielectric layers. The Lorentz medium introduces a frequency‐dependent permittivity that enables phase‐matching condition compensation under oblique incidence, suppressing angular dispersion without requiring geometric reconfiguration or external biasing [80]. Furthermore, this LPA approach enables multichannel THz PA under a unified dielectric thickness, a property not achievable with conventional Tamm‐based or metamaterial‐based designs. Compared with hyperbolic media, which often demand complex fabrication processes and strict anisotropy control, the Lorentz‐based implementation provides a simpler and more scalable route to achieving spectrally precise and angularly robust absorption. This work provides a practical route for realizing compact, tunable, and angularly robust THz PA suitable for future integrated photonic applications.

## 3 Conclusion

The study presents a PA architecture that integrates a Lorentz‐type dielectric dispersion with Tamm plasmon mode, enabling high‐efficiency wide‐angle PA in a structurally compact platform. By leveraging a phase and amplitude matching compensation mechanism, the proposed design overcomes the traditional tradeoff between angular robustness and multichannel tunability. Using a subwavelength Lorentzian top layer reduces the structural complexity and allows flexible spectral control via both material and geometric parameters. This approach offers a practical route for the realization of next‐generation PA that combine tunability, angular robustness, and device miniaturization, supporting further advancement of PA photonic systems.

## Author Contributions

**Yuxuan Zhou:** conceptualization, investigation, funding acquisition, writing – original draft, writing – review and editing, visualization, validation, methodology, software, formal analysis, supervision, data curation, project administration, resources.

## Funding

The author has nothing to report.

## Conflicts of Interest

The author declares no conflicts of interest.

## Data Availability

The data that support the findings of this study are available from the corresponding author upon reasonable request.
